# Study on the Sensing Coating of the Optical Fibre CO_2_ Sensor

**DOI:** 10.3390/s151229890

**Published:** 2015-12-17

**Authors:** Karol Wysokiński, Marek Napierała, Tomasz Stańczyk, Stanisław Lipiński, Tomasz Nasiłowski

**Affiliations:** 1 InPhoTech, 17 Słomińskiego St31, 00-195 Warszawa, Poland; mnapierala@inphotech.pl (M.N.); slipinski@inphotech.pl (S.L.); tnasilowski@inphotech.pl (T.N.); 2 IPT Safety, Ceramiczna St 8A, 20-150 Lublin, Poland; 3 Polish Centre for Photonics and Fibre Optics, Rogoźnica 312, 36-060 Głogów Małopolski, Poland; tstanczyk@pcfs.org.pl

**Keywords:** optical fiber sensors, CO_2_ sensors, gas sensors, chemical sensors, sol-gel coatings, silica gels, indicator dyes, absorption-based sensors

## Abstract

Optical fibre carbon dioxide (CO_2_) sensors are reported in this article. The principle of operation of the sensors relies on the absorption of light transmitted through the fibre by a silica gel coating containing active dyes, including methyl red, thymol blue and phenol red. Stability of the sensor has been investigated for the first time for an absorption based CO_2_ optical fiber sensor. Influence of the silica gel coating thickness on the sensitivity and response time has also been studied. The impact of temperature and humidity on the sensor performance has been examined too. Response times of reported sensors are very short and reach 2–3 s, whereas the sensitivity of the sensor ranges from 3 to 10 for different coating thicknesses. Reported parameters make the sensor suitable for indoor and industrial use.

## 1. Introduction

### 1.1. An Overview of Current Carbon Dioxide Sensors Applications

Carbon dioxide is an important gas in ventilation, agriculture and many industrial branches. The possibility of determining the concentration of CO_2_ enables one to control various processes and improve safety standards. The increase of CO_2_ level is a major concern in a majority of buildings where it may affect the living comfort and in extreme scenarios, can even cause health issues. Agriculture and greenhouses are other fields where an appropriate level of CO_2_ content in the atmosphere is crucial. Miscellaneous industries also require the monitoring of CO_2_ concentration with fermentation plants and food storehouses being the most well-known ones. The control of carbon dioxide level is also important in underground mining, especially in coal mines, in which an increase of CO_2_ concentration may be an indicator of fire or ventilation malfunction. 

In the latter application safety is of major importance. Even a small damage of an electronic device may end in a formation of spark which can lead to explosion. Therefore, all the devices brought underground need to be validated as anti-sparking ones. Optical remote sensing seems to be an attractive way of achieving this goal, since no electric current is required, and thus they are intrinsically explosion safe. Hence, the optical fibres are a promising candidate for a gas monitoring in harsh environments.

### 1.2. Commercially Available CO_2_ Sensors

Currently, there are several techniques available on the market, which are used for CO_2_ detection [1,2,3]. The most widespread carbon dioxide sensors are based on non-dispersive infrared (NDIR) detection and do not utilize optical fibres. It is noteworthy that while the measurement itself is very quick, the diffusion of gas through the protecting membrane is much slower. The installation of membrane is omitted in sensors dedicated to applications, where dust and other contaminants are not expected to be present. Capnographs do not utilize such membranes and thus provide a very short response time. Another type of commercially available CO_2_ sensors are semiconductor devices, which exhibit different resistance, capacitance or impedance at different carbon dioxide concentrations [2,3,4]. They are very cheap, however, they may suffer from cross-sensitivity to other gases and base signal shifting. Optical fibre fluorescent sensors are another emerging technology already present on the market, which will be discussed more thoroughly in the subsequent subsection.

### 1.3. Optical Fibre Gas Sensors

There are various ways of utilizing the optical fibres in various systems for chemical monitoring. They can be used for sample illumination and signal reception or they can be sensors *per se* by using special active fibre coatings, which are sensitive to the analysed substance.

The most straightforward method of chemical analysis is a measurement of the absorption. It is especially useful for measuring small concentrations of substances e.g., CO [5,6] or CH_4_ [7]. Optical fibres can be used to deliver light to the measurement chamber and subsequently to the detectors. High sensitivity of the method can be further improved by the use of dielectric mirrors in cavity enhanced spectroscopy [8,9] The absorption of chemicals can be measured by using hollow-core fibres, where the light is guided in the air inside the fibre [10]. 

The measurement of the substance concentration can also be performed by investigating the evanescent wave absorption, e.g., by microstructured optical fibres [10], D-shaped optical fibres [11] or Plastic Clad Silica (PCS) fibres. Optical fibres may also be deformed e.g., by tapering to achieve this goal [12]. Such tapers can be made by using either drawing at high temperature [13] or etching in hydrogen fluoride solution [14]. 

Refractive index may also be used for an analysis, since it may change in some polymers and silica gels when in contact with a certain chemical. This fact is widely used in humidity sensing by employing Fabry-Perot nanocavities at the tips of optical fibres [15,16]. Refractive index change can also be employed in the coated fibre tapers [17]. The chemical may be adsorbed on the surface of optical fibre, which facilitates the detection thereof [18]. There is also a class of compounds, which change their volume when exposed to an analyte e.g., water [19,20]. 

Fluorescence found many applications in sensing with optical fibres [21]. Usually, a PCS fibre or optical fibre taper or optical fibre tip is coated with a matrix material with an active substance incorporated in it. The possible matrix materials are usually chosen from polymer and sol-gel groups. Measurement of the intensity of the emission peak can be used for determination of concentration of the analysed substance [22,23]. However, such a measurement procedure requires the detection of a narrow band of light, which can be achieved by using optical spectrum analysers, but it is also very expensive. Alternatively, an optical filter can be used for detection, which, however can be costly, too. Apart from the intensity-based fluorescent sensing, decay time may also be measured in order to determine the analyte concentration, which is often called a lifetime-based method [22,23,24]. Lifetime-based sensors are currently commercially available [23]. Both luminescent methods are widely used for sensing of many parameters such as pH and the concentration of CO_2_ or O_2_ [25]. 

In a similar way, colour changing dyes can also be used for sensing purposes [23,25,26]. PCS fibres, fibre tapers, fibre tips or other structures can be coated with a polymer or silica gel containing an organic indicator dye [27]. The analyte reacts with the indicator inside the sensing layer, which changes the colour of the latter. Each form of the dye has got different absorption spectrum. Sensors based on such solutions have much simpler construction than fluorescent-based sensors. 

Another method of chemical sensing is based on the inscription of a long period fibre grating (LPFG) in the optical fibre. LPFGs are sensitive to many parameters like temperature, strain, refractive index of surrounding media and other factors [28,29]. Chemical sensors employing LPFGs must be coated with an analyte-sensitive substance [28,29].

The described methods of chemical detection can be used for monitoring different gases. Not all of them, however, are suitable for each substance. Humidity levels have been measured by detecting a change of absorption, refractive index or reflectance in interferometers, LPFGs and other structures [28,29,30,31]. 

Ammonia sensing has also been thoroughly explored. Sensors based on optical fibres usually utilize the fact that ammonia is a basic gas and it increases the pH [32,33], but other solutions have also been reported [17].

Fluorescence has been employed for the detection of oxygen [25,34,35]. Optical fibre O_2_ sensors utilize special noble metal complexes, which change their fluorescence yield when exposed to different oxygen concentrations. 

Optical fibre sensors of CO_2_ utilize the acidity of this gas. Many publications on CO_2_ sensors are focused on fluorescence [25,35,36]. Few substances, like pyranine (also known as HPTS) or fluorescein exhibit dependence between pH and fluorescence yield. Even more solutions have been reported for optical sensors not utilizing optical fibres, however, such results can be easily reproduced in the fibre optic field [24,37,38]. 

## 2. Proposed CO_2_ Sensor

The aim of the authors was to develop a low-cost, fast responsive optical fibre CO_2_ sensor. Despite the availability of a great variety of reported fluorescent CO_2_ sensors, the authors recognized that those solutions are too expensive due to the necessity of filtering the wavelengths. The absorption-based solution with indicator dye doped coating [27] seems to allow one to conduct gas sensing in a much simpler way. On the other hand, the reported sensors [27] suffer from long response times, a low sensitivity to CO_2_ and a high cross-sensitivity to humidity. 

Indicator substances usually operate only if they are dissolved in a matrix material. The most frequently encountered ones are polymers and silica gels. Manifold polymers can be used for this purpose [19,24,29,36,37,39,40,41]. Silica gels are made during a controlled hydrolysis of alkoxysilanes and their derivatives. The most frequently used substrates for silica gel preparation are tetraethoxysilane [42,43], triethoxymethylsilane [27], triethoxy-*n*-octylsilane [44] and other derivatives. A comparison of both polymers and silica gels is presented in Table 1. Due to a high porosity, a possibility of adjusting the parameters of the coating and other advantages, the authors have chosen silica gels for the preparation of gas sensors. Other, inorganic matrix materials like ZnO have also been reported [45], however, they provide less sensing possibilities. 

In the reported solution, a fragment of PCS optical fibre acrylic coating is removed and then it is recoated with silica gel containing pH sensitive dye. Indicator dyes, which can be used for this purpose include e.g., methyl red, phenol red, phenolphthalein, thymol blue. These substances change colour when exposed to environments with different pH levels. For example, methyl red changes from yellow to red when pH decreases [46], phenol red changes from fuchsia to yellow [47] and thymol blue changes from blue to yellow [48]. The ranges at which a colour changes occur are quite narrow: 4.8–6.0 for methyl red, 6.4–8.0 for phenol red and 8.0–9.6 for thymol blue [49]. The silica gel layer after solvent evaporation becomes porous, which facilitates the interaction between the dyes and carbon dioxide. Some indicators, like thymol blue, do not work properly in a form of powder or after annealing in silica gel [27]. The interaction between single molecules is responsible for this phenomenon. Molecules need to be separated to be acceptors or donors of a proton. 

**Table 1:** Comparison of the most important properties of polymers and silica gels.

	Polymers	Silica Gels
Porosity	varied: low to moderate	high
Transparency	varied: moderate to high	varied: low to high
Surface quality	smooth	pores
Mechanical behaviour	elastic	varied: moderately elastic to brittle
Cost of single deposition process	low	low
Cost of series coating process	low	moderate (periodic gelling process)
Easiness of deposition	easy	easy
Easiness of the solution preparation	easy	moderately difficult
Coatings with different thickness	possible to deposit	possible to deposit
Solubility of organic dyes	limited to those soluble in a solvent dedicated to the polymer	limited to those soluble in water and alcohols
Leakage of organic dyes	possible unless copolymerized or immobilized otherwise	possible unless immobilized

Carbon dioxide can react with water according to the following equations:

(1) $${\text{CO}}_{\text{2}}+{\text{2H}}_{\text{2}}\text{O}\to {\text{HCO}}_{\text{3}}^{\text{-}}+{\text{H}}_{\text{3}}{\text{O}}^{\text{+}}$$

(2) $${\text{HCO}}_{\text{3}}^{\text{-}}+{\text{H}}_{\text{2}}\text{O}\to {\text{CO}}_{\text{3}}^{\text{2-}}+{\text{H}}_{\text{3}}{\text{O}}^{\text{+}}$$

The formed hydronium ion may subsequently react with the indicator dye, which finally leads to a colour change. The presented mechanism requires water to be present at the reaction side. Therefore, sensors utilizing such principle of operation should not be annealed.

## 3. Experimental Section 

Carbon dioxide sensors were prepared according to the procedure described below. A 12 cm fragment of a plastic clad silica optical fibre was stripped of its acrylic coating by immersing it for 60 s in dichloromethane and subsequent manual taking off the softened polymer. Afterwards we have coated the fibre with silica gel doped with indicator dye and then we have left it for curing for 24 h. Silica gels were prepared from triethoxymethylsilane (TEMS) according to the procedure described in [27]. All the substrates, solvent and a liquid detergent were placed in a plastic vial. The reaction mixture was stirred for 6 min until a transparent liquid has been obtained. Then a stripped fragment of PCS fibre has been immersed in the prepared solution. It is possible to control the thickness of the silica gel layer by managing the fundamental solution parameters [50]. Nevertheless, since available thickness values for single dip coating process are low, in this work thicker layers have been obtained by repeating the immersion process several times. Dyes used for the sensors preparation included thymol blue, phenol red, methyl red and bromothymol blue. The analysed dyes were chosen due to their pH change range close to neutral, which corresponds to changes induced by CO_2_ gas, which decreases the pH of water from neutral to weakly acidic. After the curing time, the fibre was attached to a PMMA slide. One end of the fibre was connected to the light source and the other one was put inside a detector. For temperature tests a Peltier module was placed under the slide to control the temperature of the sensor. All the other tests were performed at a constant temperature.

The light source used for phenol red and methyl red samples was a 520 nm pigtailed laser (Thorlabs, Newton, NJ, USA). Thymol blue and bromothymol blue sensors were illuminated with 650 nm laser, which was FIS visual fault locator. The mentioned wavelengths were chosen due to big differences between acidic and basic spectra of the dyes. Every pH sensitive dye has at least two forms specific for certain type of environments (e.g., acidic and basic). Each form of the dye has a different absorption spectrum. For certain substances it is possible to choose the wavelength range, within which, the difference of absorption is high. This is evident in the analysed dyes [46,47,48,49]. That provides a possibility of working at a single wavelength instead of working with a broader spectrum of light. One just needs to choose the wavelength at which there is a big difference between the spectra of different forms of a dye. A GL55 series photoresistor (Senba Optoelectronic, Shenzhen, China) was used for the detection of light. 

The composition of an atmosphere inside the chamber was regulated by dosing pure CO_2_ from a gas pressure bottle. For reducing the concentration of CO_2_, the chamber was purged with a fresh air. As a result, the concentration of carbon dioxide decreases slowly, which makes it possible to easily record the sensor readings at different concentrations. Response time measurements were performed by a rapid filling the chamber with maximum CO_2_ gas flow and then a subsequent rapid purging by compressed air. The actual concentration of carbon dioxide inside the chamber was monitored by two commercial CO_2_ sensors based on NDIR and electrochemical principles of operation. Humidity was monitored by an electronic meter. Temperature was measured with a pyrometer to ensure that the actual temperature of the sensor was being reported.

**Figure 1:**
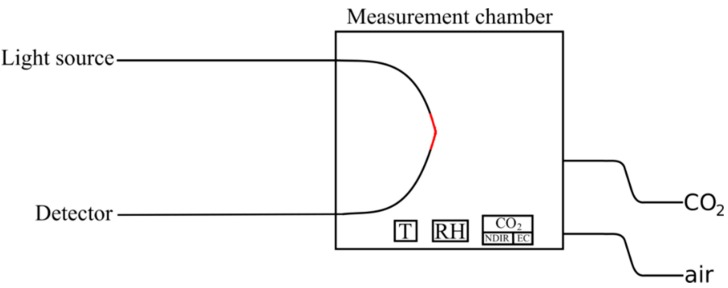
Measurement set up used for CO_2_ sensing. The red fragment represents the optical fibre sensor.

The measurements were carried out in a dedicated gas chamber, which is depicted in Figure 1.

## 4. Results and Discussion

### 4.1. Analysed Indicator Dyes

Several substances acting as CO_2_ indicators have been examined. Out of the four tested dyes—thymol blue, phenol red, methyl red and bromothymol blue—except for bromothymol blue three were responsive to carbon dioxide concentration changes in air. Bromothymol blue has already been reported as an indicator in pH and gas sensors [32,42], and the lack of activity reported by the authors can be attributed either to the interaction between the dye and a sol gel basic catalyst or to the change of pH sensitivity range due to immobilization of the dye. All the other dyes yielded CO_2_ responsive sensors. Sensors incorporating thymol blue and phenol red have already been reported in the literature, but their sensitivities were much lower [27]. An optical fibre CO_2_ sensor utilizing methyl red has not been reported yet. Making such a sensor was even claimed to be impossible due to a too low pH colour change range of this dye [27]. However, due to the decomposition of alkyl-substituted ammonia catalyst upon drying, which can take several days, the pH of the sensing layer decreases. Therefore, a sensor incorporating thymol blue, which initially has a blue active layer, turns green and subsequently turns yellow. This colour change is also associated with the decrease of the sensitivity to CO_2_. Eventually, after 5–10 days when the catalyst decomposes inside the active layer, the sensor ceases its operation and is no longer working. The authors have encountered a similar issue with phenol red. Due to the lower range of pH inducing colour change it retained little of its sensitivity after a few weeks. The opposite problem was observed for methyl red. This dye has a pH range of colour change within the weakly acidic region. Therefore, initially it was not responsive to carbon dioxide, but after decomposition of the catalyst, pH of the sensing layer decreased and then it was able to detect CO_2_ within a full range of concentrations. This is why it is possible to prepare CO_2_ sensor with methyl red dye. This is also the first time, when the stability issue for an absorption based carbon dioxide optical fibre sensor is reported and a method of circumventing thereof is proposed. 

**Figure 2:**
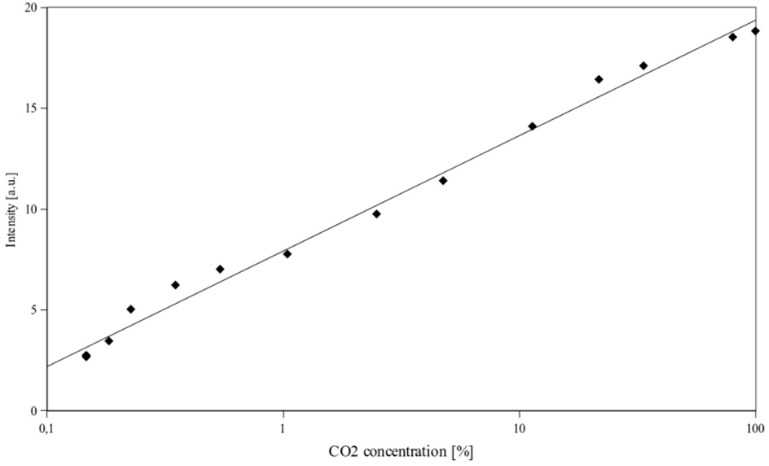
Dependence between the intensity of light transmitted through an optical fibre sensor containing thymol blue as an indicator dye and the CO_2_ concentration.

Figure 2 shows the dependence between the CO_2_ concentration and the intensity of the 650 nm laser light transmitted by the optical fibre sensor comprising thymol blue. The series of experiments have been performed 24 h after preparation of the sensor. Response of the sensor is logarithmic as a function of carbon dioxide concentration. Dependence between the intensity of transmitted light and CO_2_ concentration can be described by an equation: *I* = 2.48ln[CO_2_] + 7.9, where [CO_2_] represents the percentage of CO_2_ in air. *R*^2^ value is equal to 0.99. Character of the dependence between CO_2_ concentration and the transmitted light intensity is a result of a way, in which colour of the dye changes. As the pH of the layer gradually changes, the absorption spectrum of the layer also changes gradually. The ratio of the intensity of light transmitted at 100% CO_2_ concentration and the intensity of light transmitted at 0.1% CO_2_ concentration equals 8.3, which exceeds the value of 1.7 previously reported for this dye [27]. An increase of the intensity of transmitted light occurs along with a colour change of an active layer from dark blue to bright yellow, which is consistent with a literature spectroscopic data [48]. The response characteristics of the sensor have been deteriorating after performing the experiments. After approximately two weeks it did not respond to CO_2_ any more.

**Figure 3:**
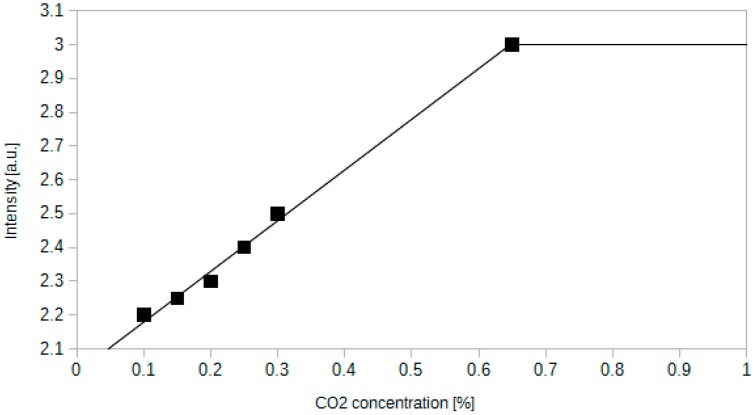
Dependence between the intensity of light transmitted through an optical fibre sensor containing phenol red as an indicator dye and the CO_2_ concentration.

The other examined sensor was based on phenol red dye. In the beginning it worked within a full range of CO_2_ concentration. The response characteristics tended to deteriorate after a few days. The results obtained two weeks after sensor preparation are depicted in Figure 3. The sensor was illuminated with a 520 nm laser light.

The response of the sensor is linear within a low CO_2_ concentration range. The dependence between CO_2_ concentration and the intensity of light transmitted by the fibre is equal to: *I* = 1.50[CO_2_] + 2.03, where [CO_2_] represents the percentage of carbon dioxide. *R*^2^ value is equal to 0.97. Sensor detects the gas only until the concentration reaches 0.65%. The ratio of maximum and minimum signal intensity equals 1.36, which is much less than for thymol blue. However, if only a 0.65% limit is taken into account, than the signal ratio is equal to 3.1 for thymol blue, which makes the difference smaller. During the operation of the sensor, the active layer changed its colour from red to pale yellow, which stands in accordance with spectroscopic data available in the literature [47]. The limit of 0.65% of CO_2_ concentration is a result of a relatively narrow pH range for which the colour of phenol red changes. As the basic catalyst decomposes, the pH of an active layer decreases, thus the available CO_2_ detection range narrows down. Taking into account the given analytical expression for the response of phenol red to carbon dioxide and the experimental uncertainty (±0.05 a.u.) one can expect that the lower limit of detection should not be higher than 0.03% of CO_2_, which is suitable for indoor use.

**Figure 4:**
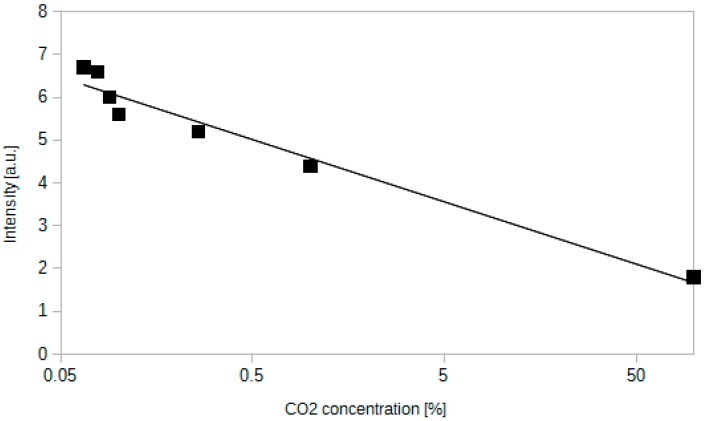
Dependence between the intensity of light transmitted through an optical fibre sensor containing methyl red as an indicator dye and the CO_2_ concentration.

The sensor containing methyl red has been operating in a stable fashion for a second week after preparation. It was tested with a laser light source working at 520 nm. Figure 4 shows the response characteristics of the sensor.

Signal of this sensor decreased when CO_2_ concentration increased, which is an opposite situation in comparison to the previous sensors. Dependence between light intensity and concentration is in this case logarithmic, similarly to the thymol blue sensor. It can be described by an equation: *I* = −0.63ln[CO_2_] + 4.58. *R*^2^ value is equal to 0.96. The quotient of the maximum and minimum intensity of the signal is equal to 10. When sensor is exposed to CO_2_, the layer changes its colour from yellow to red, which is consistent with the literature data [46]. The lowest CO_2_ concentration, which was tested was 0.08%, but concentrations down to 0.01%–0.02% should also be possible to measure, since it was reported for similar solutions [27].

### 4.2. Influence of the Dye Concentration

**Figure 5:**
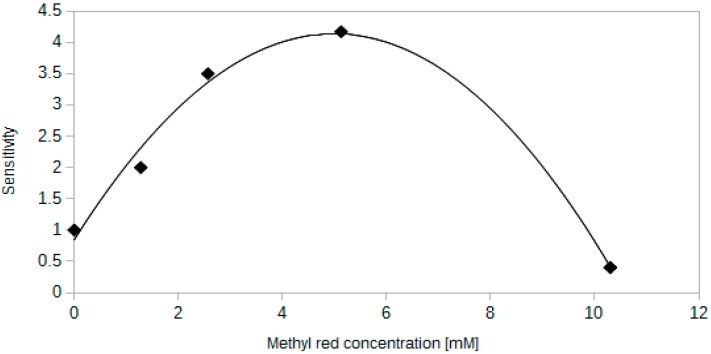
Dependence between the sensitivity and the concentration of methyl red in the sensing layer. Each sample has the same geometrical parameters and the same layer thickness.

Since the light goes through the active layer deposited on an optical fibre, the concentration of an indicator dye is crucial to the response characteristics of the sensor. It is reasonable, that the more dye is in the sensor, the stronger response should be reported. However, other factors may also affect the output signal of a sensor, which is presented in Figure 5. Figure 5 and all the other plots presented henceforth show the results obtained for methyl red-based sensors.

Sensitivity in Figure 5 refers to a ratio of the transmitted signal intensity at 0.1% of CO_2_ to the signal intensity at 100% of CO_2_. This term is present in subsequent figures and is defined in the same way.

One can notice that initially an increase of the methyl red concentration improves the sensor sensitivity. As the concentration rises, the dependence becomes less inclined and eventually goes down. This phenomenon can be explained by a limited solubility of methyl red in silica gel. The response characteristics get to the point in the dye concentration domain, when no further response increase is possible. If one would increase the amount of the dye in the active layer, the excess of this substance would form a very fine powder during the drying. This was observed as a red, opaque appearance of an active layer on optical fibre in contrast to yellow, transparent coatings for low concentrations. What is more, dye in a powdered form may behave in a different way than dissolved powder. As one can see in Figure 5, after reaching the maximum, the dependence not only decreases, but even goes below sensor response value equal to 1. This means that after reaching this point, the sensor works the opposite way *i.e.*, during the increase of the CO_2_ concentration, the intensity of transmitted light increases. The most reasonable explanation for this behaviour is that an acidic form of methyl red is more soluble in silica gel matrix than its basic form. Such a phenomenon may be peculiar to methyl red, since other dyes simply reach the maximum response at a certain concentration [27]. 

### 4.3. Influence of the Active Layer Thickness on the Response of the Sensor

**Figure 6:**
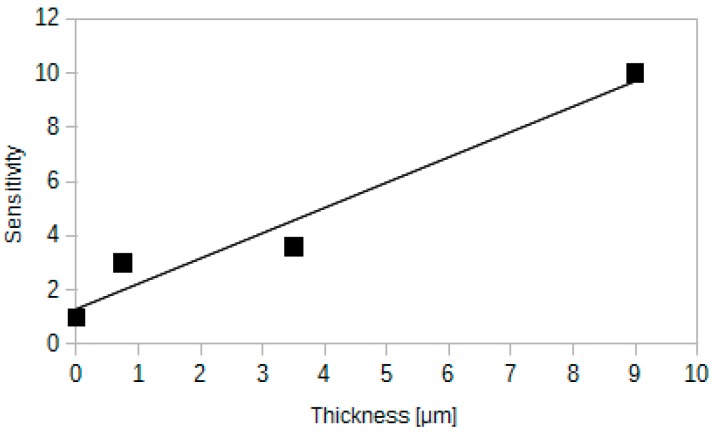
The influence of a sensing layer thickness on the sensitivity. Measurements have been performed on scanning electron microscope.

One can expect that similarly to the concentration, the thickness of an active layer should also play an important role in the sensor operation. It is possible to check the influence of thickness on the sensor signal by preparing samples with different thickness of a sensing layer. This can be achieved by deposition of different number of layers on an optical fibre. The results of such an experiment are depicted in Figure 6. The plot shows that the response of the sensor (defined the same way like in the previous subsection) rises when thickness of a sensing layer rises. 

### 4.4. Repeatability of the Sensor Response

**Figure 7:**
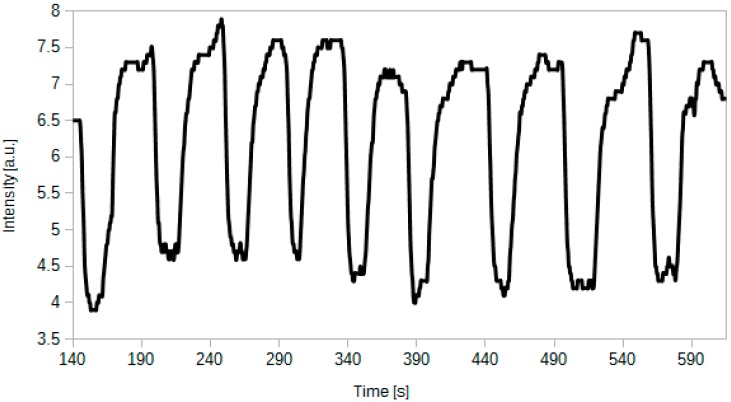
Intensity of the transmitted light for a sensor consecutively exposed to CO_2_ and fresh air with CO_2_ content below 0.1%.

Sensor aptitude for work should be tested not only during a short test, but also during a longer experiment. Figure 7 presents a plot of the intensity of light transmitted through the optical fibre sensor during the experiment. The sensor was consecutively exposed to 100% CO_2_ and fresh air. This results in a number of dips in a sensor operation time plot.

One can observe that the sensor works in a stable, repeatable way. The sequential CO_2_ exposures do not affect the base level of the intensity neither for low nor for high CO_2_ concentrations. Fluctuations present in Figure 7 are a result of a non-uniform gas distribution during those fast-paced tests. 

### 4.5. Response Time of the Sensor

**Figure 8:**
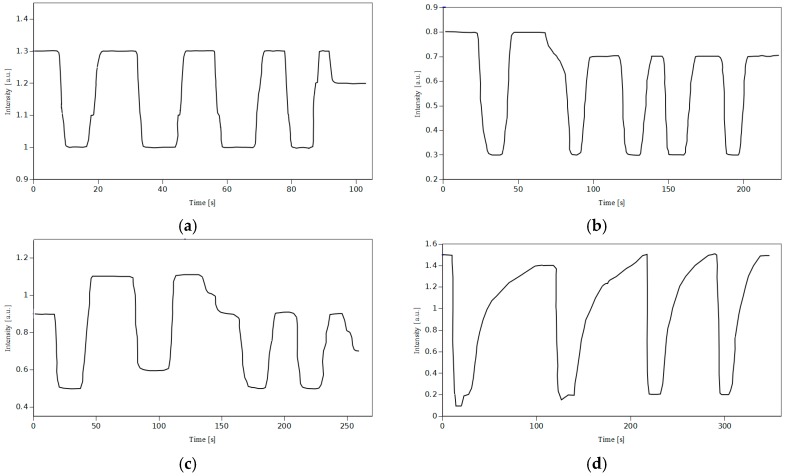
Response time characteristics for four different sensing layer thicknesses, 0.75 μm, 3 μm, 3.5 μm and 9 μm ((**a**), (**b**), (**c**), (**d**) respectively).

The most important parameter of the sensor, which affects the response time, is the thickness of the sensing layer. The thicker the layer, the more time is needed for CO_2_ to diffuse through it. Figure 8 presents response time plots for four different layer thicknesses.

**Figure 9:**
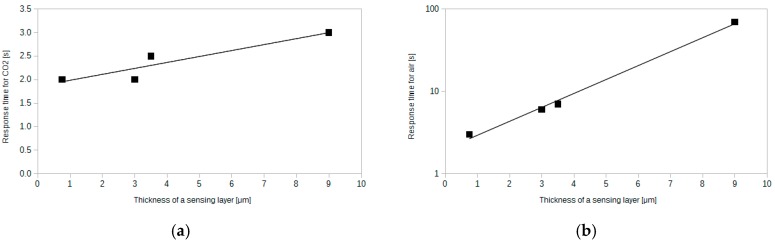
Dependence between the sensing layer thickness and (**a**) response time during exposure to 100% CO_2_ (**b**) response time during exposure to air after CO_2_ impulse.

The plots presented in Figure 8 follow the rule mentioned above. What is more, one can notice that the time needed for the sensor's response to 100% CO_2_ is much lower than the time needed to get back to the base reading after the end of the CO_2_ pulse. The difference increases significantly when the thickness increases. The response time for the exposure to CO_2_ changes slightly over the whole range of thickness. Figure 9 shows the dependence between the response time and the sensing layer thickness. 

The lowest reported response times are very short. When switching from low to high carbon dioxide concentration, the response time is equal to 2 s for 0.75 μm thick silica layer. For the same thickness, the response time during switching from high to low CO_2_ concentration is equal to 3 s. These values are much lower than for other similar sensors reported in the literature [27,30]. Such short response times make it possible to use such sensor for on-line CO_2_ monitoring. Apart from the low response times, thin layers exhibit satisfactory sensitivity (see Figure 6) equal to 3. Therefore thin layers are expected to be an optimum choice for versatile applications.

### 4.6. Influence of Temperature

**Figure 10:**
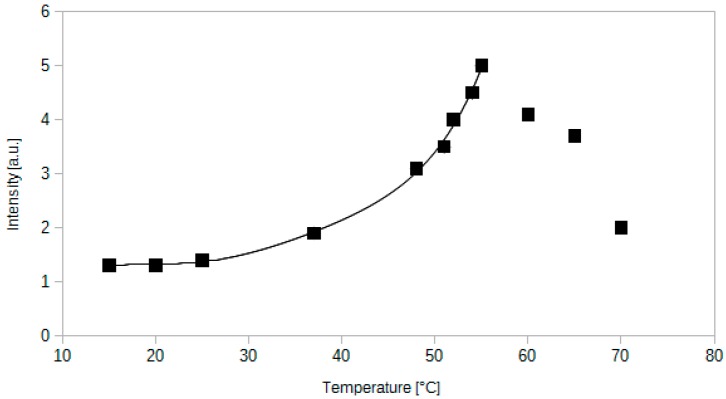
Dependence between the light intensity transmitted by an optical fibre sensor containing methyl red and the temperature. Measurement performed at 0.1% of CO_2_ in air.

Temperature is a factor which can substantially change during a measurement. Therefore, its impact on the sensor's response should be thoroughly inspected. The temperature of the sensor was controlled by Peltier module and it has been measured by a pyrometer at the centre of the optical fibre sensor. The intensity of light transmitted through the optical fibre increases when the temperature increases as it is shown in the Figure 10.

The dependence between the intensity of transmitted light and the temperature is weak below 25 °C. Above this temperature, the intensity rises significantly until reaching 55 °C–60 °C range. The intensity at the highest point (55 °C in Figure 10) is 3.85 times higher than at 20 °C. Therefore it is important to measure or control simultaneously the temperature while using such optical fibre sensor. 

The phenomenon described above can be a result of an increased solubility of the indicator dye. Another factor which could also influence the operation of the sensor could be an increased affinity to CO_2_ of the dye or silica gel.The alternative explanation for the sensor’s behaviour could be a change of the active layer refractive index. A decrease of the refractive index would increase the amount of light in the silica core.

**Figure 11:**
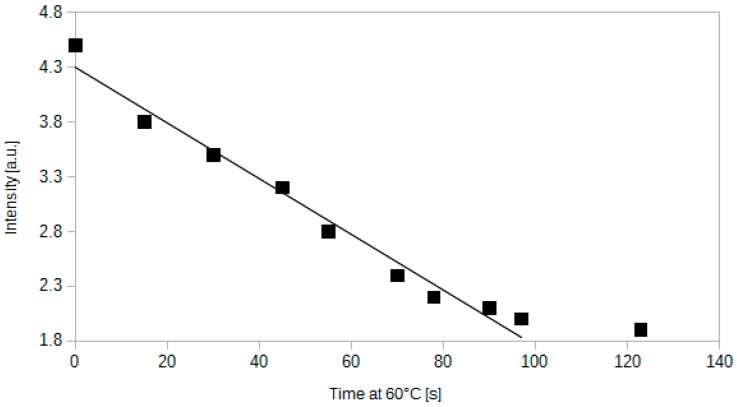
Dependence between the intensity of light transmitted through the sensor and the time after reaching 60 °C. Measurement performed at 0.1% of CO_2_ in air.

A series of thermal experiments has been performed in order to determine the temperature at which the drop in transmission occurs. It has been noted that the intensity increases until 55 °C. Then, between 55 °C and 60 °C it does not rise any more. At 60 ± 2 °C it drops abruptly and decreases by 58%. Further increase of the temperature makes the transmission even weaker (see Figure 10). The decrease of the intensity is linear in time, which is presented in Figure 11.

It is important to highlight that before reaching the temperature of 60 °C, the sensor was operational. After reaching 60 °C it did not respond to CO_2_ any more. The time needed for the whole change is approximately equal to 100 s at 0.1% of CO_2_. The reason for such an abrupt transition is probably related to the nature of methyl red indicator dye, since such behaviour has not been reported for other silica gel-based optical fibre sensors [35]. Therefore, the most probable explanation for the analysed phenomenon is that methyl red in silica gel at 60 °C losses its ability to bind hydrogen ion. This can be caused either by a decrease of humidity in the silica gel and subsequent precipitation of the dye or by molecular properties of methyl red. The decay time of 100 s is probably needed to achieve the threshold temperature for the whole optical fibre sensing region.

The described process was repeatable. However, the optical fibre regained its ability to sense carbon dioxide after cooling to temperatures lower than 60 °C. For instance, the sensor resumed operation after cooling to 43 °C, which occurred approximately 4 min after the start of cooling from 60 °C. This speaks in favour of the humidity-based hypothesis described in the previous paragraph.

It is important to notice that the drop of the transmission is non-differentiable as a function of temperature. This could indicate that a high order phase transition may occur, since the imaginary part of the dielectric constant (responsible for absorption) is non-differentiable. 

### 4.7. Cross-Sensitivity to Other Gases

**Figure 12:**
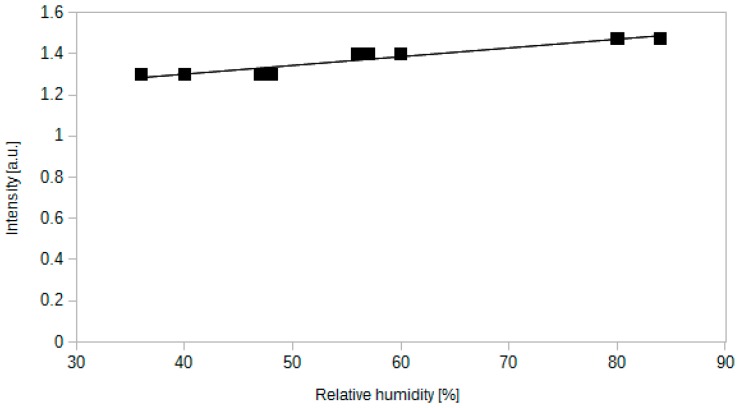
Dependence between relative humidity and the intensity of transmitted light. The measurements were carried out at 18 °C with a sensor containing methyl red.

Equations (1) and (2) indicate that the processes employing CO_2_ inside the sensing layer require water to be present at the reaction site. Thereby, humidity may also affect the operation of the sensor. We expected that the humidity dependence should also be relatively low, similar to the solution reported in [30]. The results are shown in Figure 12. 

When increasing the relative humidity from 36% to 84%, the intensity of light increases only by 13%. It is consistent with the results reported for methylene blue [30]. Measured range of relative humidity is typical for majority of applications. It is necessary to highlight the fact, that the response of the sensor to humidity can also be dependent on temperature.

Although the cross-sensitivity to water vapour is low, the sensor responds strongly to liquid water and dew. For instance, the transmitted intensity strongly increases when the sensing part of the fibre is immersed in water. Therefore during the operation the sensor should be protected against water and other liquids.

Carbon dioxide is not the only gas which can influence the pH of a sensing layer. There are several other gases, e.g., NO_2_ and SO_2_, which also react with water and produce H_3_O^+^ ions. Similarly, NH_3_ is a basic gas, which increases pH of the sensing layer. All the mentioned gases may influence the readings of the reported sensor. However, they are usually present in the atmosphere at low levels, which may be too low to affect the sensor operation. 

It is possible to design and prepare more selective sensors for solely detecting gases more acidic than carbon dioxide or more basic than this gas. For instance, a sensor similar to the reported ones, but also containing a buffer, which would keep a pH approximately around 4 would be less affected by typical fluctuations of CO_2_ and it would mostly respond to more acidic gases. However, a dye which changes its colour within lower pH values should be used for this purpose instead of methyl red or phenol red. In a similar way ammonia can also be detected. Other solutions like the use of certain polymers and not using a matrix material at all [32] have already been reported and also may help solving the problem of cross-sensitivity.

Thus a sensor matrix incorporating: (a) the reported CO_2_ sensor; (b) sensor of strongly acidic gases; (c) sensor of basic gases; (d) humidity sensor and (e) temperature sensor should be self-consistent and should provide the option of multigas sensing.

### 4.8. Final Recommended Solution

The authors have tested several sensors with different indicator dyes, different response times and different sensitivities. Thymol blue, due to its instability, was found not to be useful for further work. Phenol red yielded a sensor with 0.65% maximum limit of detection, which is suitable for ventilation monitoring. Methyl red provided a sensor which could operate at low and high CO_2_, concentrations, and therefore it is recommended dye for versatile applications. Taking into account the examined silica gel thickness, thin layers (approximately 0.75 µm) are suitable for the majority of applications, since the sensitivity reaches a value of 3 and response times are very low (2–3 s). To the best of our knowledge, these response times for thin silica gel layers are the lowest ones reported for an absorption-based optical fibre CO_2_ gas sensor. Similarly, the reported sensitivity for a thick silica gel coating is the highest for such a sensor type.

When compared to commercially available NDIR sensors, the reported sensor has much faster response time (2–3 s instead of 60 s [1]) and can be used for measuring higher CO_2_ concentrations (up to 100% instead of 0.5%–1% for NDIR). On the other hand, the resolution of NDIR devices within the low concentration range is much higher. The reported sensor has a faster response than commercially available electrochemical sensors and it has similar resolution (measurement range from 0.035% to 1% or 5% or from 0.2% to 95%, response time of several minutes [2]).

### 4.9. Self-Referencing Arrangement

**Figure 13:**
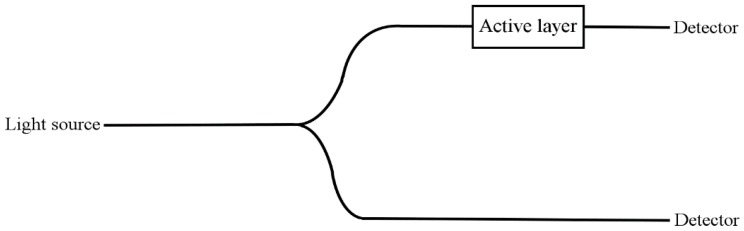
Self-referencing arrangement of the sensor. Lines represent optical fibres.

The described sensor can possibly find application in manifold areas. We propose a simple self-referencing arrangement of the sensing system, in which light from the source passes through an optical fibre coupler and then it passes independently through the sensor and the reference arm. The arrangement is shown in Figure 13.

In such a sensing system the concentration of CO_2_ would be determined from the intensity ratio of both signals passing through the sensor and the reference arm. What is more, the described sensor arrangement enables one to omit the problem of the light source power instability.

## 5. Conclusions

Broad range CO_2_ optical fibre sensors have been presented. The reported sensors are made by deposition of an active silica gel coating onto a plastic clad silica optical fibre. Considering the tested indicator dyes, methyl red was found to be the most utilitarian one, since it operates over a whole range of CO_2_ concentrations. It is noteworthy that a methyl red CO_2_ optical fibre sensor has not been reported before and it was even claimed to be impossible to manufacture one. The stability of these absorption-based carbon dioxide optical fibre sensors has been examined for the first time. A method of obtaining a stable sensor has been proposed and experimentally verified with good results. What is more, the phenol red-containing sensor also operated stably within the low range of carbon dioxide concentrations. This may destine phenol red-based sensors to indoor use and methyl red ones to industrial applications. It has been noted that an increase in the active layer thickness increases the sensitivity of the sensor, but it also substantially increases the response time. Humidity was found to have a weak influence on the response of the sensor. Reported sensors exhibit high sensitivities reaching *I*_0.1%_/*I*_100%_ of 10 for the 9 μm thick layers. For an active layer thickness of 0.75 μm the response time can be as low as 2 s for switching from low to high CO_2_ concentration and 3 s for the opposite process. The reported sensor thus exhibits much lower response time and higher sensitivity than other absorption-based solutions presented elsewhere in the literature.
